# Characterization of pediatric brain tumors using pre-diagnostic neuroimaging

**DOI:** 10.3389/fonc.2022.977814

**Published:** 2022-10-17

**Authors:** Shannon Green, Victoria D. Vuong, Paritosh C. Khanna, John R. Crawford

**Affiliations:** ^1^ Department of Radiology, University of California, San Diego, CA, United States; ^2^ Department of Pediatrics, Rady Children’s Hospital, San Diego, CA, United States; ^3^ Department of Pediatrics, Division of Child Neurology, Children’s Hospital Orange County, Orange, CA, United States; ^4^ Department of Pediatrics, University of California Irvine, Irvine, CA, United States

**Keywords:** pediatric neuroradiology, pediatric brain tumor, tumor growth, brain neoplasm, tumor growth rate, apparent diffusion coefficient

## Abstract

**Purpose:**

To evaluate for predictive neuroimaging features of pediatric brain tumor development and quantify tumor growth characteristics in patients who had neuroimaging performed prior to a diagnosis of a brain tumor.

**Methods:**

Retrospective review of 1098 consecutive pediatric patients at a single institution with newly diagnosed brain tumors from January 2009 to October 2021 was performed to identify patients with neuroimaging prior to the diagnosis of a brain tumor. Pre-diagnostic and diagnostic neuroimaging features (e.g., tumor size, apparent diffusion coefficient (ADC) values), clinical presentations, and neuropathology were recorded in those patients who had neuroimaging performed prior to a brain tumor diagnosis. High- and low-grade tumor sizes were fit to linear and exponential growth regression models.

**Results:**

Fourteen of 1098 patients (1%) had neuroimaging prior to diagnosis of a brain tumor (8 females, mean age at definitive diagnosis 8.1 years, imaging interval 0.2-8.7 years). Tumor types included low-grade glioma (n = 4), embryonal tumors (n = 2), pineal tumors (n=2), ependymoma (n = 3), and others (n = 3). Pre-diagnostic imaging of corresponding tumor growth sites were abnormal in four cases (28%) and demonstrated higher ADC values in the region of high-grade tumor growth (p = 0.05). Growth regression analyses demonstrated R^2^-values of 0.92 and 0.91 using a linear model and 0.64 and 0.89 using an exponential model for high- and low-grade tumors, respectively; estimated minimum velocity of diameter expansion was 2.4 cm/year for high-grade and 0.4 cm/year for low-grade tumors. High-grade tumors demonstrated faster growth rate of diameter and solid tumor volume compared to low-grade tumors (p = 0.02, p = 0.03, respectively).

**Conclusions:**

This is the first study to test feasibility in utilizing pre-diagnostic neuroimaging to demonstrate that linear and exponential growth rate models can be used to estimate pediatric brain tumor growth velocity and should be validated in a larger multi-institutional cohort.

## Introduction

Central nervous system tumors are the most common type of solid tumor in the pediatric population and are the leading cause of cancer-related mortality in childhood (1). Noninvasive characterization of brain tumors has rapidly evolved in the past five years as neuroimaging techniques have advanced. For example, various magnetic resonance imaging (MRI) techniques have been used to discriminate tumor type, including apparent diffusion coefficient (ADC) value (2, 3) mass spectroscopy (4), magnetic resonance fingerprinting (5), perfusion imaging, and dynamic susceptibility contrast (6, 7). Neuroimaging also has been used to assess growth characteristics of common brain tumors in adults, including high-grade gliomas (8–12) and meningiomas (13–15). In children, growth patterns of even the most common pediatric brain tumors (juvenile pilocytic astrocytoma, medulloblastoma, ependymoma) have not been formally studied. Furthermore, no study has characterized pediatric brain tumors based on neuroimaging features before diagnosis to date.

There are potential advantages of evaluating pre-diagnostic neuroimaging. First, suspicious features can be critically evaluated. Since it is possible to distinguish pediatric brain tumor subtypes based on MRI characteristics (16), presumably the same diagnostic features used to classify brain tumors can be assessed on pre-diagnostic imaging in the region of subsequent tumor growth, e.g., enhancement, perfusion, ADC values. With increasing use of advanced diagnostic imaging and evolving imaging techniques, more opportunities exist to identify tissue at risk for tumor development.

Secondly, tumor growth patterns can be evaluated on pre-diagnostic neuroimaging. Thus far, the most widely accepted adult tumor growth models include (1): exponential growth (i.e., constant volume doubling time) (2); linear growth (i.e., constant radial growth velocity); and (3) Gompertzian growth (i.e., sigmoid function), which assumes progressively decreased volume doubling time due to diminishing tumor nutrients. Of the three models, the Gompertzian model is most supported, with previous studies demonstrating a growth plateau (8, 13). However, no consensus for which model best estimates tumor growth exists secondary to the complexity and multifactorial nature of tumor development. For example, tumors with the same histopathology may have different growth characteristics based on molecular biomarkers (12), size at diagnosis (8, 14), aggressiveness (13), patient age (13), tumor location (14), and tumor region measured (12). Furthermore, few studies have assessed early tumor growth patterns because tumors clinically present with progressive symptomatology, typically when macroscopic, limiting knowledge of the exact start of tumor development. By evaluating imaging before the brain tumor diagnosis, tumor characteristics, including growth pattern, can be further elucidated.

This study aimed to evaluate pediatric neuroimaging both before and at the time of brain tumor diagnosis to identify possible predictive imaging features for tumor development and to evaluate pediatric brain tumor growth patterns.

## Methods

A retrospective review of 1098 consecutive pediatric patients with a brain tumor diagnosed between January 2009 and October 2021 at Rady Children’s Hospital-San Diego were reviewed. Inclusion criteria included patients ≤ 18 years old with cross-sectional neuroimaging (e.g., CT, MRI) before and at the time of tumor diagnosis. Patients with known cancer syndromes were excluded. University of California San Diego Institutional Review Board approval (IRB 800824) was obtained on 8/25/2021. Informed consent of legal guardians of patients was waived due to the retrospective design.

### Clinical information

Demographic information was obtained, including gender, race, age, indication at time of neuroimaging, modality (i.e., CT, MRI), clinical presentation, and time interval between imaging studies. Patient management course and clinical outcome were recorded, including surgical, chemotherapy, and/or radiation. If applicable, degree of resection was noted.

Histopathologic diagnosis and corresponding current World Health Organization (WHO) classification were recorded whenever possible (17). High-grade tumors and low-grade tumors were differentiated based on WHO grade and were analyzed separately. Cases of ependymoma were classified as low-grade or high-grade based solely on WHO grading as molecular classification was not available.

### Neuroimaging features

All initial diagnostic MRI examinations were performed on GE scanners (GE Healthcare, Chicago IL). The majority (n=14) of initial diagnostic scans were performed on 1.5 Tesla scanners, with one scan performed on a 3 Tesla scanner. Protocols included a sagittal and axial T1 without contrast, axial FLAIR, T2, Diffusion with ADC maps, axial GRE, SWI or SWAN sequence and three plane T1 post-contrast sequences, either three plane spine-echo post-contrast T1 sequences, or a combination of axial T1 spin-echo sequence and three plane FSPGR gradient T1 “brain-lab” sequences. One initial exam (performed on the 3T scanner) included single voxel MR Spectroscopy as well. Imaging of the entire spine without and with contrast was performed in nine patients to screen for metastatic disease.

All images were viewed and evaluated using the IBM Watson Health Merge PACS™ software, v7.1.2.154108. Tumor neuroimaging characteristics were recorded, including location, enhancement, total tumor size, and solid component size. All brain MRIs performed following diagnosis and before treatment were reviewed, including four low-grade tumors initially managed by observation. Total tumor volume was estimated using a general ellipsoid formula (V = 4/3π*a*b*c), where V represents the volume and the a, b, and c variables represent half the tumor diameter in transverse, anteroposterior, and craniocaudal dimensions, respectively. The observed growth rates of each tumor by diameter and solid tumor volume were calculated using difference in tumor size between pre-diagnostic and diagnostic imaging divided by the time interval between imaging studies.

**Figure 1:**
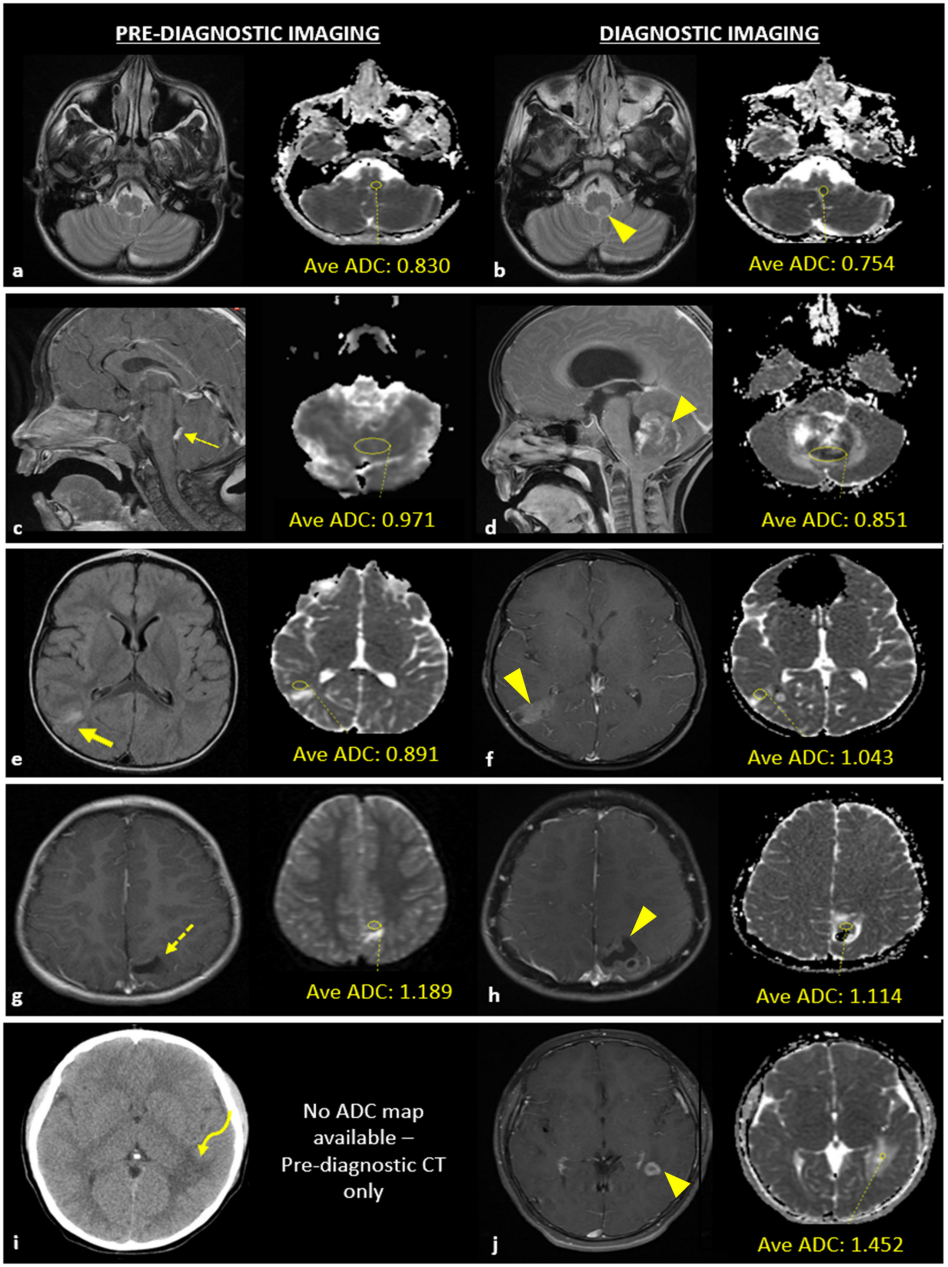
Example of apparent diffusion coefficient (ADC) acquisition for a patient with a case of central nervous system lymphoma **(A, B)** and examples of neuroimaging studies with abnormal findings at the site of pediatric brain tumor development **(C–J)**. **(A)** T_2_-W MRI sequence of pre-diagnostic neuroimaging without a macroscopic tumor present and corresponding ADC map with a yellow circle that delineates the area used to calculate average ADC value of the tissue region in which a brain tumor would later develop; **(B)** T_2_-W MRI sequence obtained at the time of diagnosis demonstrating the macroscopic lesion (arrowhead) and corresponding ADC map with a yellow circle that delineates the area of the tumor used to calculate average ADC value. **(C)** Pre-diagnostic contrast enhanced (CE) -T_1_WI sagittal image showing thickened, enhancing choroid (thin arrow) and pre-diagnostic ADC map; **(D)** post-diagnostic CE-T_1_WI sagittal image and ADC map of atypical teratoid rhabdoid tumor (ATRT). **(E)** Pre-diagnostic T_2_/FLAIR-WI axial image showing hyperintense T_2_/FLAIR signal (thick arrow) and pre-diagnostic ADC map; **(F)** post-diagnostic CE -T_1_WI axial image with ADC map of low-grade glioma. **(G)** Pre-diagnostic CE -T_1_WI axial image showing encephalomalacia/gliosis (dashed arrow) and pre-diagnostic ADC map; **(H)** post-diagnostic CE -T_1_WI axial image with ADC map of a ganglioglioma. **(I)** Pre-diagnostic axial CT image showing white matter hypoattenuation (curved arrow); no pre-diagnostic ADC map was available due to lack of MR imaging; **(J)** post-diagnostic CE-T_1_WI axial image with ADC map of JXG.

Mean ADC values were measured using ellipsoid regions of interest (ROI). ROIs were drawn on the largest area of regions of solid and/or enhancing tumor on the diagnostic MRI and the corresponding region on pre-diagnostic imaging (**Figure 1A**). ADC values were not available for six pre-diagnostic imaging studies, due to lack of MRI imaging (n = 4) or due to intraventricular tumor location (n = 2). Diffusion restriction could not be evaluated in two diagnostic neuroimaging studies due to susceptibility artifact.

The neuroimages were reviewed by a senior neuroradiologist (PK) and senior neuro-oncologist (JC) with more than 30 years combined experience in interpreting neuroimaging in pediatric brain tumors. The inter reader agreement of the ROI’s were 100% in the unblinded limited series.

### Statistical methods

Parametric data were expressed as mean[range] and compared using the Student’s t-test. Nonparametric data were expressed as mean[range] and compared using the Welch’s t-test. F-test was used to determine variance. All tests were 2-sided and were determined to be significant if the *p-*value ≤ 0.05. The atypical teratoid/rhabdoid tumor (ATRT) was separated from tumor growth analyses and used as a reference for high-grade tumor growth rate given macroscopic tumor was visualized on the pre-diagnostic study retrospectively. The case of osteoblastic osteosarcoma was also excluded from growth analyses given tumor origination outside the craniospinal axis.

To model tumor growth for high- and low-grade tumor groups, best linear and exponential models were fit to plots of tumor size versus time interval between pre-diagnostic and diagnostic imaging studies, using diameter and solid tumor volume for size, respectively. Goodness of fit (R^2^) of each model was recorded. The y-intercept was set at zero for the linear growth model to represent lack of visible tumor on the pre-diagnostic imaging.

Statistical analysis was performed using Excel^®^, v2110 (Build 14527.20276 Click-to-Run).

## Results

Fourteen total patients (1%) met inclusion criteria (**Table 1**). Mean patient age at the time of diagnostic imaging was 8.1[2.8-14.5] years. Tumor types included ATRT (n = 1), ependymoma (n = 3), medulloblastoma (n = 1), low-grade glioma (n = 3), pineoblastoma (n = 1), pineocytoma (n = 1), osteoblastic osteosarcoma (n = 1), CNS lymphoma (n = 1), ganglioglioma (n = 1), and juvenile xanthogranuloma (JXG) (n = 1). Seven tumors were classified as high-grade tumors (Cases 1-7) and seven were classified as low-grade tumors (Cases 8-14) based on WHO grading. Mean interval time between pre-diagnostic and definitive diagnostic imaging studies for all cases was 3.0[0.2-8.7] years. The mean interval time between studies was not statistically significant for high-grade versus low-grade tumors (1.8[0.2-3.1] years vs. 4.5[1.4 – 8.7] years, respectively, p = 0.1, **Table 1**). Notably, three low-grade gliomas managed by observation demonstrated no interval growth in size during the observation period, up to 2.5 years.

**Table 1:** Patient Demographic and Clinical Information.

Case	Gender	Race	PRE-DIAGNOSTIC TIMEPOINT				Interval Between Imaging Studies^€^ (Years)	DIAGNOSTIC TIMEPOINT			Management
			Age* (Years)	Study Ordered	Clinical Setting	Imaging Indication		Clinical Setting	Imaging Indication	Diagnosis	
1	male	white	1.6	brain MRI	outpatient	strabismus, nystagmus	2.4	ED	vomiting, fever	ATRT^a^	surgery (GTR); chemotherapy; RT; stem cell rescue
2	male	white	13.6	brain MRI	ED	headache	0.2	inpatient	incidental (follow up ETV)	CNS lymphoma^a^	surgery (NTR)
3	female	white	7.5	brain MRI	inpatient	worsening headaches	0.2	ED	AMS, follow up extra-axial collections	Osteoblastic osteosarcoma^a^	chemotherapy
4	female	white	1.3	brain MRI	outpatient	involuntary movements	2.6	ED	vomiting, HA, ataxia	Medulloblastoma^a^	surgery (GTR); chemotherapy
5	female	not specified	11.2	sinus CT	outpatient	noncontributory (allergic rhinitis)	2.0	ED	chronic headache, double vision	Supratentorial ependymoma (WHO Grade 3)^a^	surgery (GTRx2); proton radiation
6	female	white	2.5	brain MRI	unknown (OSF)	unknown	2.1	ED	vomiting	Pineoblastoma^a^	surgery (STR); proton radiation; chemotherapy
7	male	black	3.6	brain MRI	outpatient	noncontributory (autism workup)	3.1	inpatient	headache, right facial droop	Supratentorial ependymoma (WHO Grade 3)^a^	surgery (NTR –> NTR –> GTR); chemotherapy; RT
8	female	not specified	11.8	brain MRI	outpatient	involuntary head movements	2.7	outpatient	various neurological complaints	Posterior fossa ependymoma (WHO Grade2)^b^	surgery (GTR); RT
9	female	white	0.7	brain MRI	outpatient	seizures	2.1	outpatient	incidental (work up syringohydromyelia)	Pineocytoma ^b^	surgery (GTR)
10	female	white	1.2	head CT	unknown (OSF)	headache	3.7	outpatient	headache	Low-grade glioma^b^	observation
11	male	white	2.3	brain MRI	inpatient	incidental (cardiac transplant workup)	8.7	inpatient	seizures	Low- grade glioma^b^	surgery (GTR)
12	female	white	1.1	head CT	ED	incidental (trauma)	4.5	inpatient	seizures	Low-grade glioma^b^	observation
13	male	white	3.0	brain MRI	outpatient	leg clonus and right Babinski	6.5	inpatient	seizures	Ganglioglioma^b^	surgery (STR) w/recurrence (GTR/NTR)
14	male	white	9.5	head CT	ED	incidental (trauma)	1.4	inpatient	seizures	Juvenile xanthogranuloma^b^	biopsy/observation

*Mean age of patients with high vs low grade tumors was not significantly different at the pre-diagnostic timepoint (p = 0.47) nor diagnostic timepoint (not provided in table, p = 0.99).

^€^Mean interval time between studies for high vs low grade tumors was not significantly different (p = 0.10).

a high-grade tumor reference.

b low-grade tumor reference. AMS, altered mental status; ATRT, atypical teratoid rhabdoid tumor; CNS, central nervous system; ED, emergency department; ETV, endoscopic third ventriculostomy; GTR, gross total resection; HA, headache; MRI, magnetic resonance imaging; NTR, near total resection; OSF, outside facility; RT, radiation therapy; STR, subtotal resection.

For high-grade tumors, the most common clinical presentation prompting diagnostic neuroimaging was headache/vomiting (5 of 7 cases). For low-grade tumors, seizure was the most common clinical presentation (4 of 7 cases). Neurologic complaints (e.g., involuntary movements, nystagmus) comprised the majority remaining clinical presentations. Two patients were diagnosed with tumors for incidental reasons (i.e., trauma workup, autism workup) and both measured less than 1.5 mL in volume at diagnosis. Notably, a higher proportion of the diagnostic neuroimaging studies were obtained as emergent studies (79%) compared to the pre-diagnostic neuroimaging (36%).

Neuroimaging features of the tumors are described in **Figure 1**; **Table 2**. Four cases (28%) demonstrated abnormal findings at the site of subsequent tumor growth on pre-diagnostic imaging. Of these cases, one demonstrated thickened, enhancing tissue that was initially thought to represent prominent choroid given the size and location which ultimately progressed leading to a diagnosis of ATRT (**Figure 1C**). The remaining three cases were pathologically diagnosed as low-grade and demonstrated nonspecific findings on the pre-diagnostic neuroimaging at the site of subsequent tumor growth, including T2/FLAIR signal abnormality (**Figure 1E**), encephalomalacia (**Figure 1G**), or nonspecific white matter attenuation (**Figure 1I**). Pre-diagnostic MRI was not available in four cases (28%).

**Table 2:** Pre-diagnostic and Diagnostic Neuroimaging Features.

Case	Retrospective Evaluation of Pre-diagnostic Imaging	ADC Value (10^-3^ mm^2^/s)	Tumor Location	Enhance-ment	Restricted Diffusion	ADC Value (10^-3^ mm^2^/s)	Tumor Size, Max Dimension^€^ (cm)	Solid Tumor Volume^€^ (mL)	Tumor Volume^€^ (mL)	Disseminated Disease at Diagnosis?	Diagnosis
1	enhancing, thickened choroid	0.971	vermis	yes	yes	0.851	5.1	29.1	29.1	no	ATRT^a^
2	no MR evidence of tumor	0.830	pontomedullary	yes	yes	0.754	1.1	0.5	0.5	no	CNS lymphoma^a^
3	no MR evidence of tumor	0.884	multifocal supratentorial	yes	yes	0.549	2.6	6.9	13.6	yes^c^	Osteoblastic osteosarcoma^a^
4	no MR evidence of tumor	0.821	4th ventricle	yes	yes	0.730	4.3	32.3	32.3	no	Medulloblastoma^a^
5	limited visualization; no midline shift	N/A (CT)	frontal lobe	yes	susceptibility artifact	N/A	6.5	31.1	98.5	no	Supratentorial ependymoma (WHO Grade 3)^a^
6	no MR evidence of tumor	N/A (IV)	pineal	yes	susceptibility artifact	N/A	3.2	6.0	6.0	yes^d^	Pineoblastoma^a^
7	no MR evidence of tumor	0.826	occipitoparietal region	yes	yes	0.421	9.1	42.9	207.6	no	Supratentorial ependymoma (WHO Grade 3)^a^
8	no MR evidence of tumor	N/A (IV)	4th ventricle	yes	no	0.813	0.9	0.1	0.1	no	Posterior fossa ependymoma (WHO Grade 2)^b^
9	no MR evidence of tumor^e^	1.602	pineal	yes	no	1.635	1.3	0.4	0.4	no	Pineocytoma^b^
10	no CT evidence of tumor	N/A (CT)	pons	no	no	1.275	1.7	1.5	1.5	no	Low-grade glioma^b^
11	T2 signal abnormality	0.891	occipital lobe	yes	no	1.043	2.5	4.3	4.3	no	Low-grade glioma^b^
12	no CT evidence of tumor	N/A (CT)	4th ventricle	no	no	1.516	2.1	1.4	1.4	no	Low-grade glioma^b^
13	encephalomalacia/dysplasia	1.189	parietal lobe	no	no	1.114	3.5	1.6	13.5	no	Ganglioglioma^b^
14	hypoattenuating white matter	N/A (CT)	temporal lobe	yes	no	1.452	1.3	0.9	1.1	no	Juvenile xanthogranuloma^b^

^€^ = No significant difference between high- and low-grade tumor maximum diameter (p = 0.10), solid tumor volume (p=0.06), nor total tumor volume (p = 0.16)

a High-grade tumor reference.

b Low-grade tumor reference.

c Multiple intracranial metastases present at time of diagnosis.

d Diffuse metastatic leptomeningeal spinal spread at time of diagnosis.

e No pineocytoma was present on pre-diagnostic imaging, but a separate dorsal cervicomedullary junction mass was present.

ADC, apparent diffusion coefficient; ATRT, atypical teratoid rhabdoid tumor; CNS, central nervous system; CT, computed tomography; IV, intraventricular MRI, magnetic resonance imaging.

N/A, not available.

**Figure 2:**
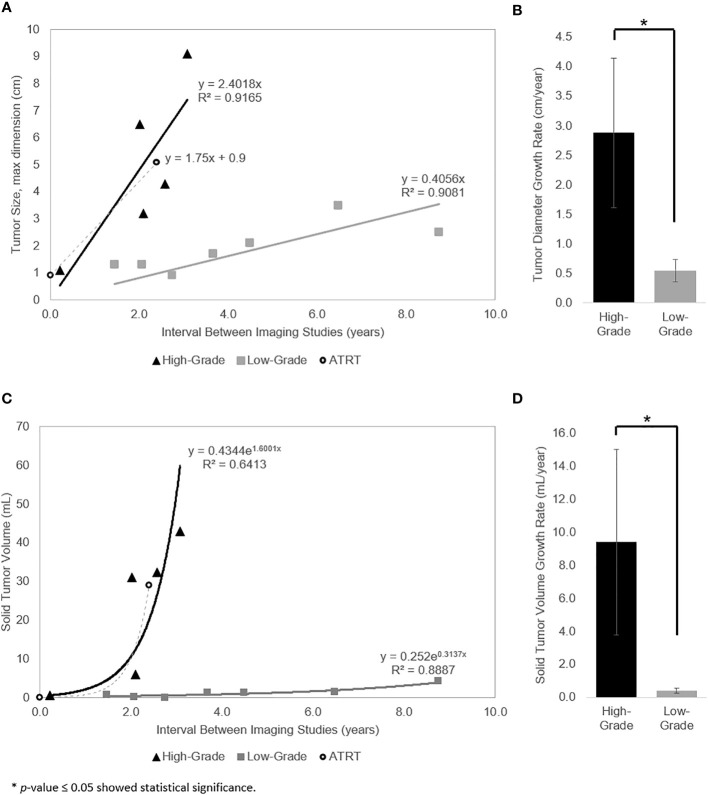
**(A)** Linear regression model for growth of the measured diameter length. Tumor diameter as a linear function of the time interval between pre-diagnostic and diagnostic imaging for high-grade tumors (triangle), low-grade tumors (square), and ATRT reference (circle). **(B)** Tumor diameter growth rate of the high- vs low-grade tumors is significantly different (p = 0.02). **(C)** Exponential regression model for growth of the measured solid tumor volume (i.e., constant volume doubling time). Calculated solid tumor volume as an exponential function of the time interval between pre-diagnostic and diagnostic imaging for high-grade tumors (triangle), low-grade tumors (square), and ATRT reference (circle). **(D)** Solid tumor volume growth rate of the high- vs low-grade tumors is significantly different (p = 0.03).

The linear and exponential regression models are represented in **Figures 2A, C**, respectively. The linear model had an excellent goodness of fit for high- and low-grade tumors (R^2^ = 0.92 and R^2^ = 0.91, respectively). The exponential model demonstrated a reasonable goodness of fit for high-grade tumors (R^2^ = 0.64) and excellent goodness of fit for low-grade tumors (R^2^ = 0.89). For the high-grade tumor reference, the velocity of diameter expansion (VDE) was 1.8 cm/year (**Figure 2A**) and the volume doubling time was approximately 500 days (**Figure 2C**). The high-grade tumors demonstrated an average VDE of 2.4 cm/year (**Figure 2A**), reflecting the minimum VDE for high-grade tumors, and estimated volume doubling time of 158 days (**Figure 2C**). The low-grade tumors demonstrated a minimum VDE of 0.4 cm/year (**Figure 2A**) and volume doubling time of 806 days (**Figure 2C**). Of note, a low-grade ependymoma with neuroimaging studies following diagnosis and before treatment showed a VDE of 0.4 cm/year during the imaging period. The average growth rate of the high-grade tumors based on diameter and solid volume were significantly higher than low-grade tumors (p = 0.02 and p = 0.03, respectively) (**Figures 2B, D**).

**Figure 3:**
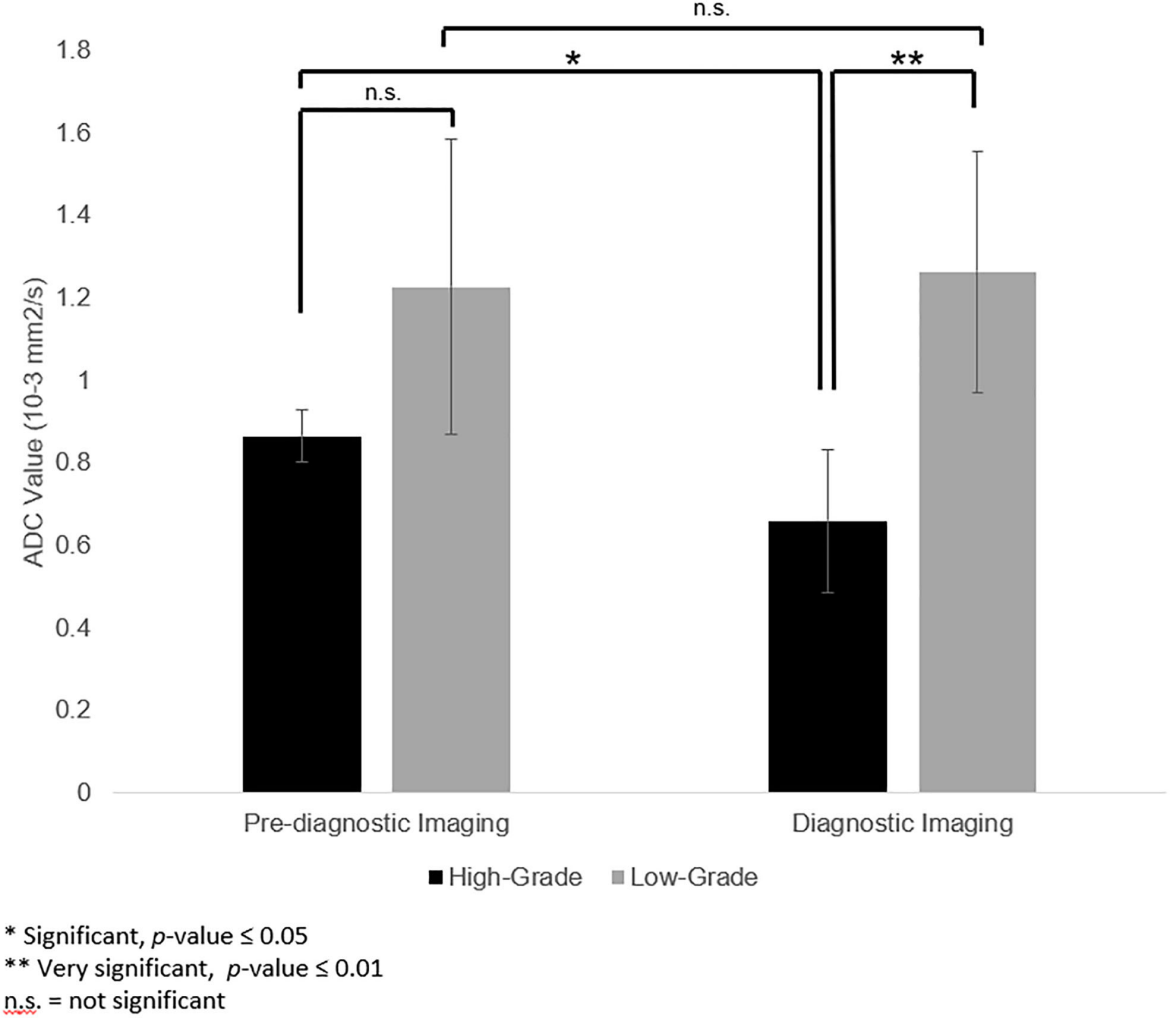
Apparent diffusion coefficient (ADC) values in the corresponding pre-diagnostic regions of tumor growth compared to ADC values of the tumor at the time of diagnosis. ADC values of high-grade tumors were lower on diagnostic imaging than the corresponding region on pre-diagnostic imaging (p = 0.05). For low-grade tumors, ADC values for low-grade tumors were not significantly different between pre-diagnostic and diagnostic imaging (p = 0.87). On pre-diagnostic imaging, there was no significant difference between ADC values of high- and low-grade tumors (p = 0.22). However, at time of diagnosis, the ADC values for the high-grade tumors was lower than the low-grade tumors (p = 0.002).

Mean ADC values of the high- and low-grade tumors on pre-diagnostic and diagnostic imaging are provided in **Figure 3**. Mean ADC value was 1.264[0.813 - 1.635 x 10^−3^] mm^2^/s for low-grade tumors and 0.661[0.421 - 0.851 x 10^−3^] mm^2^/s for high-grade tumors on diagnostic imaging. The ADC values of the high-grade tumors on diagnostic imaging were significantly lower than the corresponding region on pre-diagnostic imaging (p = 0.05), but similar on pre-diagnostic and diagnostic imaging for low-grade tumors (p = 0.87). On pre-diagnostic imaging, the ADC values were similar between the high- and low-grade tumors (p = 0.22), but on diagnostic imaging, high-grade tumors had lower ADC values than low-grade tumors (p = 0.002).

## Discussion

This study is the first to evaluate both the incidence and characteristics of pre-diagnostic neuroimaging of pediatric brain tumors. The primary goal of this retrospective study of 14 high- and low-grade pediatric brain tumors was to identify possible predictive neuroimaging characteristics at the earliest potential time point using pre-diagnostic imaging, including growth rates. Evaluation of high-grade and low-grade tumor growth demonstrated an excellent fit using the linear growth rate regression model, with R^2^ = 0.91 – 0.92 for both tumor subtypes. Moreover, pre-diagnostic imaging allowed specific evaluation of ADC values, which was not significantly different between pre-diagnostic and diagnostic imaging for low-grade tumors and was higher in pre-diagnostic imaging compared to diagnostic imaging for high-grade tumors.

Quantitative studies of pediatric brain tumor growth are lacking. Multiple factors contribute to the sparsity of data on this topic, including a relatively lower incidence of pediatric brain tumors compared to the adult population, increased awareness of radiation safety for the pediatric population, and special considerations for pediatric MRI. Particularly, high-grade tumor growth is difficult to characterize given they are often managed immediately following diagnosis, limiting long-term treatment-naïve observation. Several studies have assessed the growth characteristics of common high-grade tumors in adults (8–12). For example, Ellingson et al. found that median volumetric doubling time of preoperative, treatment-naive high-grade gliomas was 21.1 days (8). In addition, Fan et al. demonstrated an estimated VDE of 7.0 cm/year for glioblastomas and 5.1 cm/year for all high-grade gliomas (12). Stensjøen et al. reported large variations in glioblastoma growth rates, with a median velocity of radial expansion (VRE) of 3.0 cm/year. The authors also noted that one-third of tumors doubled in volume between the diagnostic and preoperative scans, while another one-third were unchanged or decreased in volume (10). Wang et al. also reported large variations in glioblastoma growth rates *in vivo* but demonstrated a VRE of 3.0 cm/year (11).

In this study, the estimated VDE was 2.4 cm/year, lower than previously reported values in the adult population. Given the exact time of initial tumor development is unknown, this calculated VDE is likely lower than the true VDE, partially accounting for the relatively slower growth rates compared to previously reported data. Another possible explanation is that the true growth rate model instead mimics the Gompertzian growth curve. If so, previously reported values would have been calculated using tumor sizes after they had passed the inflection point on the Gompertzian growth curve, falsely elevating the reported growth rate values. Finally, it is possible that tumor growth rate may be inherently slower in the pediatric population – a theory supported by the high-grade tumor reference, which demonstrated a VDE of 1.8 cm/year.

Conversely, previously reported low-grade growth characteristics are limited. Contemporaneous studies focus predominantly on analysis of adult low-grade tumor growth, including meningiomas and low-grade gliomas and demonstrate a wide range of growth patterns and rates (13–15). For example, Nakasu et al. reported volume doubling times of meningiomas ranging between 111 days and 91,400 days. Additionally, the authors noted the fastest growth rates in atypical meningiomas, intermediate growth rates in benign, noncalcified meningiomas, and slowest growth rates in calcified meningiomas. In comparison, the estimated volume doubling time in our study was 806 days, which was comparable to the intermediate growth rates presented by Nakasu et al. (15). Given the excellent fit of low-grade tumor group to the linear growth model, the estimated minimum VDE calculated in this study, 0.4 cm/year, likely better estimates the true growth rate for low-grade tumors, further supported by analysis of the posterior fossa WHO grade 2 ependymoma growth rate after diagnosis but before treatment, also 0.4 cm/year.

Though tumor growth rates have only been described in the adult population thus far, ADC values have been used to characterize brain tumors in both pediatric and adult populations. For example, Novak et al. focused on the classification of pediatric brain tumors using ADC values. The authors showed characteristic ADC values for ependymomas were 1.126 ± 0.155 × 10^−3^ mm^2^/s and 0.870 ± 0.154×10^−3^ mm^2^/s for medulloblastomas (3). Similarly, Abdulaziz et al. demonstrated ADC values ranging between 0.225–1.240 x 10^−3^ mm^2^/s for ependymal tumors, 0.107–1.571 × 10^−3^ mm^2^/s for embryonal tumors, 0.5220–0.7840 × 10^−3^ mm^2^/s for other astrocytic tumors, and 0.1530–0.8160 × 10^−3^ mm^2^/s for meningiomas (2). Yet these prior studies have not used pre-diagnostic imaging to characterize brain tissue at the site of subsequent tumor growth. In this study, the high-grade tumors demonstrated ADC values compatible with previously reported values.

While our study did not demonstrate a lower mean ADC value in the pre-diagnostic neuroimaging studies of the high-grade tumors compared to the low-grade tumors, there was a small sample size in each tumor subtype, limiting analysis and the ability to draw definite conclusions about the utility of pre-diagnostic ADC values in characterizing brain tumors. Future studies with a larger sample size may further validate or negate this point.

As demonstrated in our series, pediatric patients rarely receive neuroimaging before diagnosis of a brain tumor, limiting full characterization of different tumor subtypes. Thus far, adult tumor subtypes have been characterized using growth rate models and ADC values of neuroimaging already demonstrating macroscopic tumor. Prior studies do not evaluate ADC values of the corresponding brain tissue on pre-diagnostic imaging. This study is the first to demonstrate that both linear and exponential growth rate models can be used to estimate the growth rate of pediatric brain tumors. In addition, this study identified the potential utility of ADC values to characterize high- and low-grade tumor subtypes in both pre-diagnostic and diagnostic imaging.

This study has several limitations. The classification of low and high-grade tumors based upon WHO grade may be seen as arbitrary and not an accurate predictor of growth velocity in the absence of molecular tumor classification of tumors that was not studied in this series. Moreover, the pre-diagnostic and diagnostic neuroimaging was retrospectively analyzed and subject to the pitfalls of all retrospective analyses. Additionally, despite demonstrating an absence of macroscopic tumor on pre-diagnostic neuroimaging, the precise timing of initial microscopic tumor appearance could not be determined utilizing the available MRI sequences, limiting the determination of the exact rate of tumor growth.

The small sample size and heterogeneous mix of tumor types also results in several limitations. First, statistical evaluation of growth characteristics for each tumor type was limited. In addition, comparison of the three most common growth models could not be performed given the small sample size, limiting the ability to draw firm conclusions about the fit to a specific growth rate model. Particularly, the Gompertzian growth model could not be tested because only two studies (pre-diagnostic and diagnostic) were consistently obtained and there was an absence of macroscopic tumor on the pre-diagnostic study in most cases. Future studies with larger samples sizes of combined cohorts could further validate the accuracy of the fit to a particular growth model.

Moreover, although the imaging at time of diagnosis was uniform in terms of high quality and technique, consistent uniform neuroimaging modality including magnetic field strength, quality, and technique of pre-diagnostic images could not be achieved because some neuroimaging studies were performed at outside facilities. Notably, about a quarter of the pre-diagnostic imaging studies did not include MRI, which is more sensitive for detection of small tumors. If available, MRI could have provided more quantitative information on tumor growth.

Lastly, tumor segmentation software was not available at the study site. Therefore, tumor volumes were estimated using the ellipsoid model formula, and tumor ADC values were estimated using a best-fit ellipsoid ROI function tool within the imaging system. This prevented more exact evaluation of the tumors. Future studies with tumor software segmentation may be used to better estimate tumor volumes and ADC values in a larger multi-institutional cohort.

## Conclusion

Evaluation of pre-diagnostic neuroimaging at sites of subsequent pediatric brain tumor growth demonstrated distinct growth characteristics in high- versus low-grade tumor subtypes using both linear and exponential growth rate models. Characterization of the tumor subsets on pre-diagnostic imaging demonstrated similar ADC values of high-grade tumors compared to low-grade tumors. Larger multi-institutional cohorts are needed to better characterize pre-diagnostic MRI features that may be indicative of the earliest signs of brain tumor development.

## Data availability statement

The raw data supporting the conclusions of this article will be made available by the authors, without undue reservation.

## Ethics statement

The studies involving human participants were reviewed and approved by University of California San Diego. Written informed consent from the participants’ legal guardian/next of kin was not required to participate in this study in accordance with the national legislation and the institutional requirements.

## Author contributions

JC performed the review of all patients do determine those patients with prior neuroimaging. JC, SG, VV, and PK reviewed the series of neuroimages of those patients with prior neuroimaging. SG and VV created the linear and exponential regression models of the neuroimages sequences and performed the statistical analysis. All authors participated in the vetting of the data and preparation of the manuscript. All authors have approved this manuscript for submission.

## Conflict of interest

The authors declare that the research was conducted in the absence of any commercial or financial relationships that could be construed as a potential conflict of interest.

## Publisher’s note

All claims expressed in this article are solely those of the authors and do not necessarily represent those of their affiliated organizations, or those of the publisher, the editors and the reviewers. Any product that may be evaluated in this article, or claim that may be made by its manufacturer, is not guaranteed or endorsed by the publisher.

## Abbreviations

ADC, apparent diffusion coefficient; AMS, altered mental status; ATRT, atypical teratoid rhabdoid tumor; CE, contrast enhanced; chemo, chemotherapy; CNS, central nervous system; DLBCL, diffuse large B cell lymphoma; DX, diagnosis; ED, emergency department; ETV, endoscopic third ventriculostomy; GTR, gross total resection; HA, headache; JXG, juvenile xanthogranuloma; LGG, low-grade glioma; MRI, magnetic resonance imaging; NTR, near total resection; OSF, outside facility; RT, radiation therapy; STR, subtotal resection; VDE, velocity of diameter expansion.
